# The Impact of Climate Trends on a Tick Affecting Public Health: A Retrospective Modeling Approach for *Hyalomma marginatum* (Ixodidae)

**DOI:** 10.1371/journal.pone.0125760

**Published:** 2015-05-08

**Authors:** Agustín Estrada-Peña, José de la Fuente, Tamara Latapia, Carmelo Ortega

**Affiliations:** 1 Department of Animal Pathology, Faculty of Veterinary Medicine, Miguel Servet 177, 50013, Zaragoza, Spain; 2 SaBio, Instituto de Investigación en Recursos Cinegéticos IREC-CSIC-UCLM-JCCM, 3 Ronda de Toledo s/n, 13005, Ciudad Real, Spain; 3 Department of Veterinary Pathobiology, Center for Veterinary Health Sciences, Oklahoma State University, Stillwater, Oklahoma, 74078, United States of America; The Johns Hopkins University School of Medicine, UNITED STATES

## Abstract

The impact of climate trends during the period 1901–2009 on the life cycle of *Hyalomma marginatum* in Europe was modeled to assess changes in the physiological processes of this threat to public health. Monthly records of temperature and water vapour at a resolution of 0.5° and equations describing the life cycle processes of the tick were used. The climate in the target region affected the rates of the life cycle processes of *H*. *marginatum*: development rates increased, mortality rates in molting stages decreased, and the survival rates of questing ticks decreased in wide territories of the Mediterranean basin. The modeling framework indicated the existence of critical areas in the Balkans, central Europe, and the western coast of France, where the physiological processes of the tick improved to extents that are consistent with the persistence of populations if introduced. A spatially explicit risk assessment was performed to detect candidate areas where active surveys should be performed to monitor changes in tick density or persistence after a hypothetical introduction. We detected areas where the critical abiotic (climate) and biotic (host density) factors overlap, including most of the Iberian peninsula, the Mediterranean coast of France, eastern Turkey, and portions of the western Black Sea region. Wild ungulate densities are unavailable for large regions of the territory, a factor that might affect the outcome of the study. The risk of successfully establishing *H*. *marginatum* populations at northern latitudes of its current colonization range seems to be still low, even if the climate has improved the performance of the tick in these areas.

## Introduction

The inexorability of the changing climate and the threats it may pose by affecting the rates of transmission of tick-borne pathogens have encourage researchers to evaluate the adaptive potential of health-threatening arthropods in a changing climate [1–3]. The spatial distribution of organisms has often been modeled by linking presence—absence data with sets of climate and/or topographic variables (e.g., temperature, rainfall, fragmentation of the habitat), commonly known as abiotic variables, to capture the so-called abiotic niche and project it onto the geographical space. This is called correlative modeling, and it produces a spatial estimation of the similarity of the abiotic variables relative to the values recorded for the known distribution of the organism [4–6]. Therefore, it is an evaluation of the spatial abiotic suitability of the studied organism.

Ticks are important parasites that affect domestic and wild animals and also have an impact on public health [7–9]. Ticks are strict haematophagous arthropods and play an important role in the transmission of a number of parasites, bacteria, and viruses [10,11]. The tick life cycle comprises three active stages, each one feeding on a different host in most species. Some species may have a one- or two-hosts life cycle, molting while attached to the host [10]. Once fed, ticks molt in the relatively protected environment of the ground. Development (molting) rates are governed by temperature, whereas mortality rates are driven primarily by water availability, although some authors pointed out the effect of water availability on small differences recorded for development periods [12,13]. While questing for hosts, ticks may lose water at different rates. Thus, the complex life cycle of ticks results from a combination of interactions, including an array of hosts for immature and adult stages, geographic variability in the host range, climate trends, and alterations in landscape and vegetation that affect the weather [14].

*Hyalomma marginatum* is a tick restricted to large areas of the Mediterranean basin and Middle East [15,16]. The larvae and nymphs feed on small- and medium-sized mammals (e.g., hares, hedgehogs, and rodents) and ground-feeding birds [17]. Adults feed on larger mammals such as livestock and wild ungulates [18]. The long duration of tick attachment to host during the immature stages (12–26 days) [19] enables passive transport by migrating birds over long distances [20]. Records of ticks on migratory birds have been reported [20,21] and this causes concern about the persistence of the introduced ticks under suitable climatic conditions. The movement of ungulates infested with adult *H*. *marginatum* is also a potential route for tick spreading, provided that the abiotic conditions are adequate to support permanent tick populations [21]. In its distribution range, *H*. *marginatum* is the main vector of Crimean-Congo haemorrhagic fever virus (CCHFV), but other species of the genus *Hyalomma* are involved in the virus transmission in other areas of Africa and Asia [14]. CCHFV is the second most widespread of all medically important arboviruses after dengue and belongs to the genus *Nairovirus* in the family Bunyaviridae [22]. It causes a potentially severe disease in humans with clinical cases reported mainly in eastern Mediterranean region and central Asia, although the virus has been recorded in ticks in countries of the western Mediterranean like Morocco and Spain [15]. The virus circulates in nature in a tick—vertebrate—tick cycle [14]. Humans are normally infected through either the bite of an infected tick or the direct contact with a host or host tissues infected with CCHFV during the acute phase of infection. Therefore, from a European perspective, it is important to gain information on the geographic areas that are suitable for the main tick vector in the region and how abiotic suitability has changed based on historical records of climate.

Correlative modeling has been preferred for capturing the climate niche of ticks, and a few studies have addressed the probable changes in the range of some tick species according to regional or continental climate trends [23]. However, correlative modeling does not allow examining the role played by each climatic variable on tick’s life cycle process. Instead, modeling tick life cycle processes has been proposed as the most appropriate tool for inferring the response of tick populations to the weather [24] because the impact of each abiotic variable on every process can be evaluated. However, the complex life cycle of ticks has difficult the development of versatile models for assessing the relative impact of weather on its life cycle [25–28], something that has been partially addressed by the use of the next generation matrix and evaluation of the population-level basic reproductive ratio (R_0_) [29]. This index indicates the ability of a disease to invade and spread through a naive population [30] and has been used to describe the tendency of a population towards extinction or permanency based on the balance between reproductive and mortality processes [29,31]. Consequently, R_0_ determines the threshold values at which the balance of the tick population changes from persistence to extinction. Nevertheless, calculation of R_0_ for ticks needs accurate hosts densities, something that is missing when the methods are applied to large areas.

A model describing the life cycle development and mortality rates of *H*. *marginatum* has been built using data obtained in the laboratory under a range of controlled temperature and humidity conditions, parameterized, and evaluated [32]. Such a model has been used to track the expected impact of climate trends on the circulation of some pathogens transmitted by this tick [33] and to complement previous studies [14] on the ability of the tick to disseminate in Europe through the introduction by migratory birds from Africa [34]. This model captures the development rate of the tick, the mortality during these periods, and a general estimation of the survival of questing stages in the absence of host contacts as a function of time, temperature, and water availability. The model for *H*. *marginatum* is not intended to calculate R_0_ or estimate tick densities, because reliable estimates of host abundance cannot be obtained for large regions. Tick mortality rates during the periods of host questing depend on the availability of hosts and the lack of reliable estimates prevents its estimation. However, the life cycle model of *H*. *marginatum* is able to objectively calculate the development and mortality rates of the tick under a range of climate conditions, which renders a basic framework suitable for baseline evaluations of the effects of climate on the critical parameters of the tick's life cycle. A similar approach was used in previous research focused on the impact of climate on the basic development and mortality processes in ticks [35].

We used this modeling framework to retrospectively assess the changes in the development and mortality rates of *H*. *marginatum* in Europe during the period 1901–2009. The assessment was built on a climate dataset of temperature and humidity with a spatial resolution of 0.5° at monthly intervals [36]. The trends in the tick’s physiological processes (development and mortality rates) were evaluated in Europe, where claims about an expansion of range [37] and increased abundance [38] have been reported.

## Material and Methods

### Model development

We used an existing model aimed to capture the life cycle of *H*. *marginatum* in its simplest possible terms [32]: interstadial development rates, including oviposition, incubation, and nymphal-adult molt, and mortality rates specific to each stage and state (questing, feeding, and engorged). The model expresses the probability of ticks in each stage to advance to the next stage and the mortality associated with that life cycle event. Briefly, ticks from laboratory colonies were used to obtain basic parameters defining development and density-independent mortality rates. The original colony was formed with 168 flat adults collected at different locations in Spain (41.86°N, 0.78°W; 39.95°N, 4.11°E), Tunisia (36.51°N, 8.78°E), Italy (37.41°N, 13.64°E) and Morocco (33.81°N, 6.79°W). The tick F1 generation was the result of random mating of these adults; F2 and F3 generations were used for model development. Giant New Zealand Albino rabbits were used for tick feeding. Laboratory animals were housed and handled in accordance with the European Communities Council Directive of 24 November 1986 (86/609/EEC) and with local laws and regulations.

Tick development was modeled as a daily function of Temperature (T) and water vapour deficit (VD) with day-degree and VD summations. The VD had a small but perceptible impact on the variations of the development rates, which are commonly governed only by temperature and has been therefore included in the equations. Although relative humidity is commonly used to evaluate the mortality of tick development stages, the use of VD is preferable because it defines better the environmental constraints of the tick in its environment [13] and that variable has already been used in the development of life cycle simulation models for ticks [39] Temperature was varied between 2°C and 32°C, at steps of 5°C, and VD between 2 and 26 hPa at steps of 3 hPa. These variations include a range of unrealistic conditions, which were used only to capture the upper and lower limits of the life cycle processes. Furthermore, not every combination of T and VD was assayed, because high VD values cannot be obtained at low temperature. Engorged females were placed in groups of 10 ticks to estimate the duration of pre-oviposition, oviposition and incubation. Fifty engorged nymphs were used for each combination of environmental variables to estimate nymph to adult developmental rates. Mortality of females, eggs and molting nymphs was estimated with the same groups as before, using the same combination of T and VD. Ticks were examined daily to check for completion of development.

Daily per-capita rates of tick survival were calculated with groups of 200 unfed larvae or 50 unfed adults each, 10 days after hatching or molting, and kept at constant T and VD regimes in the laboratory. T varied from 10°C to 35°C and VD between 2 and 25 hPa. As mentioned before, this includes some unrealistic values, and not every combination of T and VD was assayed. Mortality was checked at 5, 15, 30, 45, 60, 75 and 90 days. In all cases, observations were stopped at 100% mortality.

All data were fitted to linear equations describing the effects of T and VD on developmental rates, mortality of developing stages and survival rates of questing stages (see S1 Text). A sensitivity analysis was performed in the original model development [32].

### Modeling application

**Fig 1:**
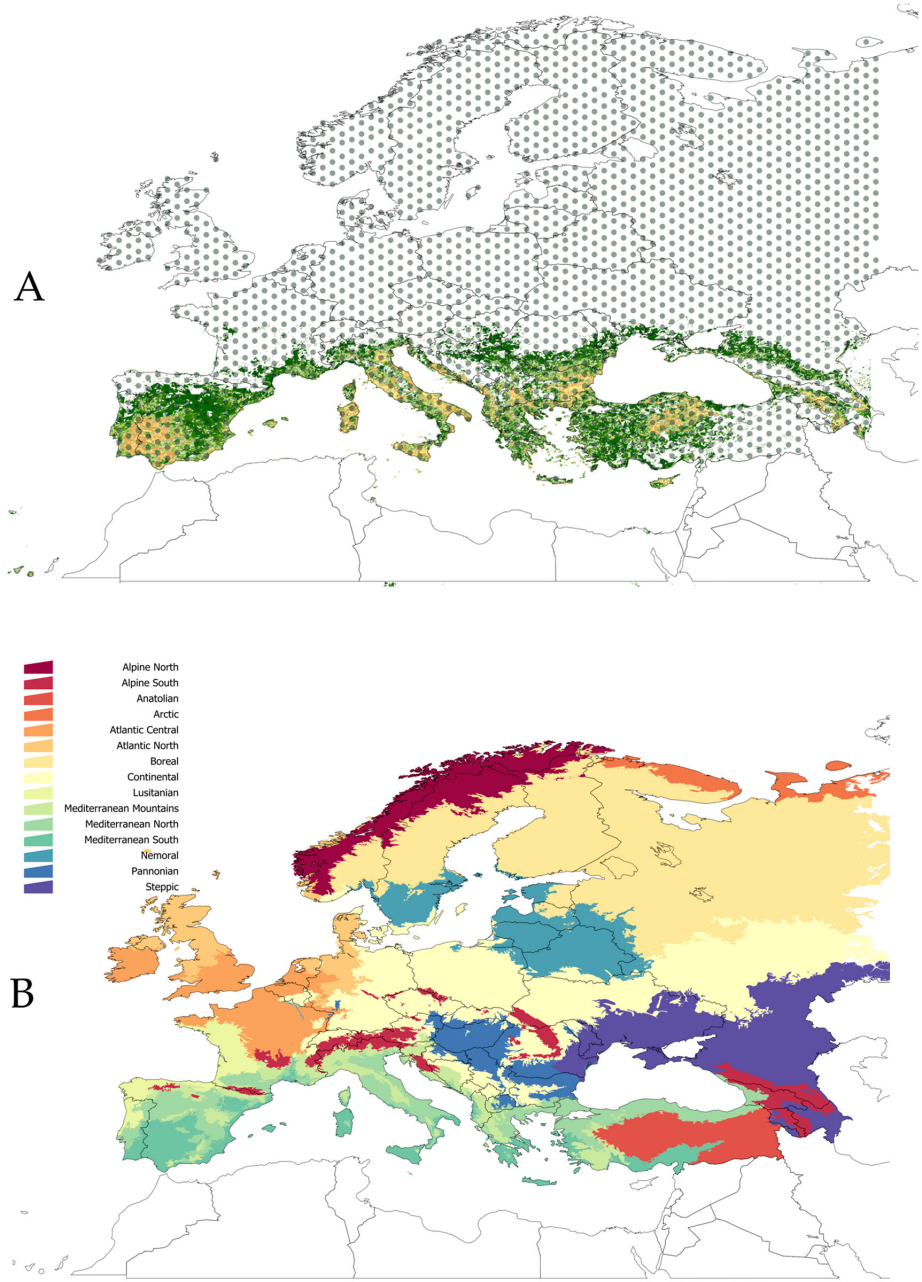
Definitions of the territory covered in this study. (A) The target territory of this study overlaid with the spatial distribution of the points for which weather during the period 1901–2009 was used as an input for further modelling analyses. Historically, *H*. *marginatum* has been collected in the coloured territory. (B) The division of the territory into climate domains as obtained from the LANMAP framework. The study is restricted to Europe. Therefore the recorded distribution of the tick in northern Africa has been disregarded.

We applied the equations described above to obtain the development and mortality rates of *H*. *marginatum* in the period between the years 1901 and 2009 in the target territory (see S2 Text). Climate data were obtained from the Climate Research Unit (CRU, University of East Anglia, UK) and included data on temperature and relative humidity collected from climate stations and interpolated to a common temporal and spatial framework (CRU TS 2.1) [36]. The dataset covers the entire world with a time resolution of one month and spatial resolution of 0.5°. For this study, we selected a territory from 69°N-10°W (top left corner) to 71°N-49°E (bottom right corner), which covers Europe. We explicitly focused on this territory because it includes the complete distribution range of the tick in its European Mediterranean range [32], as well as other territories where the tick has been found occasionally [37,40,41]. While the tick species is well known and commonly reported in northern Africa, the focus of this research was in Europe because of the concerns of ticks spreading to northern latitudes in this region and the unavailability of standard landscape classifications for spatial disaggregation of the data (see below) for northern Africa. The geographic background is displayed in Fig 1 with points marking the centre of each 0.5° grid cell for which weather conditions are available. The monthly resolution of the original dataset was converted to 10 days intervals by simple spline interpolation using the R development framework [42].

An annual development rate (DR) was calculated as the average of 10-days values given by equations describing development. The index is a value between 0 and 100, and is proportional to the development rates of the tick in one complete year. We also calculated an annual mortality rate for developing stages (MRD) as the average of 10-day values given by equations describing such features. We also calculated a survival rate for questing stages (SRQ) as the average of 10-days values. The later indices have values between 0 and 100 and are proportional to the mortality in the development periods or the survival of the different questing stages, respectively. We decided to produce separate approaches for MRD and SRQ instead of calculating them as a single index because climate exerts differential regulation of the mortality in development and questing processes [30].

### Classifications of the territory according to physiological processes

Our results were summarized based on two spatial disaggregation of the target territory. Statistical analyses of the trends of DR, MRD, or SRQ were conducted separately on two spatial domains, the first representing the area where permanent populations of *H*. *marginatum* have been recorded in the last 30 years (the "positive zone", PZ) and the second representing the area where no permanent populations of the tick have been recorded during the same period of time (the "negative zone", NZ). The analysis was intended to examine if the two zones had different rates of change for the tick's physiological processes during the study period. The basic spatial divisions were drawn from data in the literature [43] (Fig 1A).

A second spatial disaggregation was carried out according to a standard denomination of ecological domains in the target territory [44]. LANMAP is a landscape classification of Europe with several hierarchical levels, using data on climate, altitude, parent material and land use as determinant factors. LANMAP has 350 landscape types at the most detailed level. We used the first level of hierarchy, which shows the main climate domains in the target territory (Fig 1B). The purpose was to capture the trend of the rates of change of the physiological processes of the tick according to these standard ecoclimatic classifications in the target territory, in order to determine if the trends of changes were different according to the ecosystems of the territory.

We further interpreted the trends of changes of the tick physiological processes at time slices in the period 1901–2009. The aim was to categorize the raw data derived from DR, MRD, and SRQ, and derive a simple spatial ranking of the effects of the climate on the tick at different time slices. However, there is not a direct way to translate the raw values of the model equations into the threshold of mortality and development that limits the permanent populations of the tick. The physiological meaning of DR, MRD, and SRQ was drawn from the reported distribution of the tick and the range of climate suitability from a previous correlative model [32]. This allocated ranges of DR, MRD, and SRQ values into a set of categories: 1 (maximum tick performance, highest probability of presence) to 5 (minimum tick performance, highest probability of absence). The tick has been reported in categories 1 and 2, corresponding also with high values of climate suitability in correlative modeling. We therefore assumed that these categories represent optimal and suboptimal conditions for the persistence of the tick. Permanent populations of the tick have been never reported in sites of categories 4 and 5, and minimum values of the correlative modeling have been detected for these categories. These categories were assumed to represent the worst conditions for tick survival. Category 3 has intermediate characteristics and was assumed to represent a set of climate conditions where the tick is in its physiological limit. The optimum number of categories was confirmed with a discriminant analysis, using the Akaike information criterion: smaller or larger number of categories produced discriminant functions with less predictive power. Table 1 includes the complete details of the split of the physiological processes of the tick into categories. Analyses during short periods of time hardly revealed a consistent pattern, obscured by the background noise usually present in any biological data. The interval of 109 years was divided into five periods of 21 years: 1901–1922, 1923–1944, 1945–1966, 1967–1988, and 1989–2009. Each interval of time was classified with the criteria from the discriminant analysis over the 21 years averaged values of DR, MRD and SRQ.

**Table 1:** Discrimination of five categories according to the development, mortality survival, and mortality of *Hyalomma marginatum* in the target territory and classified as the reported presence/absence of the tick and climate suitability.

Category	Presence/absence	Climate (correlative) suitability	Development rate	Mortality rate of developing stages	Survival rate of questing stages
1	+	>80	18.7 ± 1.4	22.6 ± 0.7	70 ± 11
2	+	40–80	14.7 ± 1.2	24.3 ± 0.6	51 ± 11
3	-	10–40	11.4 ± 0.8	25.7 ± 0.6	29 ± 17
4	-	1–10	9.0 ± 0.8	26.8 ± 0.4	16 ± 15
5	-	<1	5.8 ± 1.1	27.9 ± 0.4	7 ± 5

The values of the correlative suitability range from 0 to 100. Values of development, mortality of developing stages and survival of questing stages are yearly averaged values and range from 0 to 100.

### Risk assessment

We sought to detect the zones where the largest changes in the physiological rates of the tick have been recorded and that overlap with zones of high host availability. The trends of faster development and lower mortality over a given threshold are coherent with a pattern of greater population turnover. Even in a suitable climate, ticks cannot persist in the absence of key hosts that are often the main determinants of tick population density. If areas of suitable climate overlap spatially with territories of great host availability, a high risk of either the persistence of newly introduced ticks or severe overpopulation should be expected. This analysis was intended to detect candidate areas for carrying out active tick surveys to either regularly check for possible invasive events or supervise changes in the density of an already established population.

This analysis has two basic steps, namely exposure and vulnerability. Exposure is interpreted here as the climate (abiotic) suitability for the tick: the exposure of a territory is higher as the performance of the tick in the climate of such an area increases. Tick exposure is directly proportional to the DR and SRQ and inversely proportional to the MRD. We considered exposure to be proportional to the categories outlined in Table 1. Vulnerability represents the biotic part of the tick’s life cycle and is proportional to the availability of suitable hosts, which permits the development of the tick. Sites where high exposure and vulnerability overlap represents zones where the requirements for tick survival and rapid turnover are met. Vulnerability depends on the presence and abundance of vertebrate hosts [45]. In the case of *H*. *marginatum*, empirical data [14,22] indicate that ruminants are the preferred hosts for tick adults. In addition, the highest turnover of the tick population is recorded in the passage from engorged females to larvae because of the high conversion rate for one female generating thousands of potentially surviving larvae [24]. Therefore, we considered the density of ruminants as the factor regulating vulnerability to this tick in the target territory. Data on the density of domestic ungulates in the target territory is available from the FAO ("Gridded distribution of livestock in the World") and maps are available at a resolution of 1 sq km (http://www.fao.org/ag/againfo/resources/en/glw/home.html, accessed December, 2011). It is well known that *H*. *marginatum* also feed on a wide range of wild ungulates [33]. However, the estimations of the spatial density of wild ungulates are only partially available for Europe and thus this part of the risk assessment can only be produced on the expected abundance of domestic ruminants. The inclusion of the distribution and abundance of wild ruminants could change these calculations.

We developed a simple relationship between exposure and vulnerability, producing a classification of the territory from 0 (minimum) to 1 (maximum). We explored and defined the fuzzy logic rules governing the link between exposure and vulnerability according to published methods [14]. The relationships include the following minimum thresholds: 7 hosts/sq km, 0.0024 annual increase in the DR, 0.001 annual decrease in the MRD, and 0.0069 annual increase in the SRQ. The combination of the minimum values resulted in a combined fuzzy value of 0; the combination of the maximum values of these variables resulted in a value of 1 with linear growth. We divided the range of fuzzy values into five equal intervals and categories, namely “very low” (0–0.2), “low” (0.2–0.4), “medium” (04–0.6), “high” (0.6–0.8), and “very high” (0.8–1). This classification reflects the combination of critical values for survival and tick population fitness based on both abiotic and abiotic variables.

## Results

**Fig 2:**
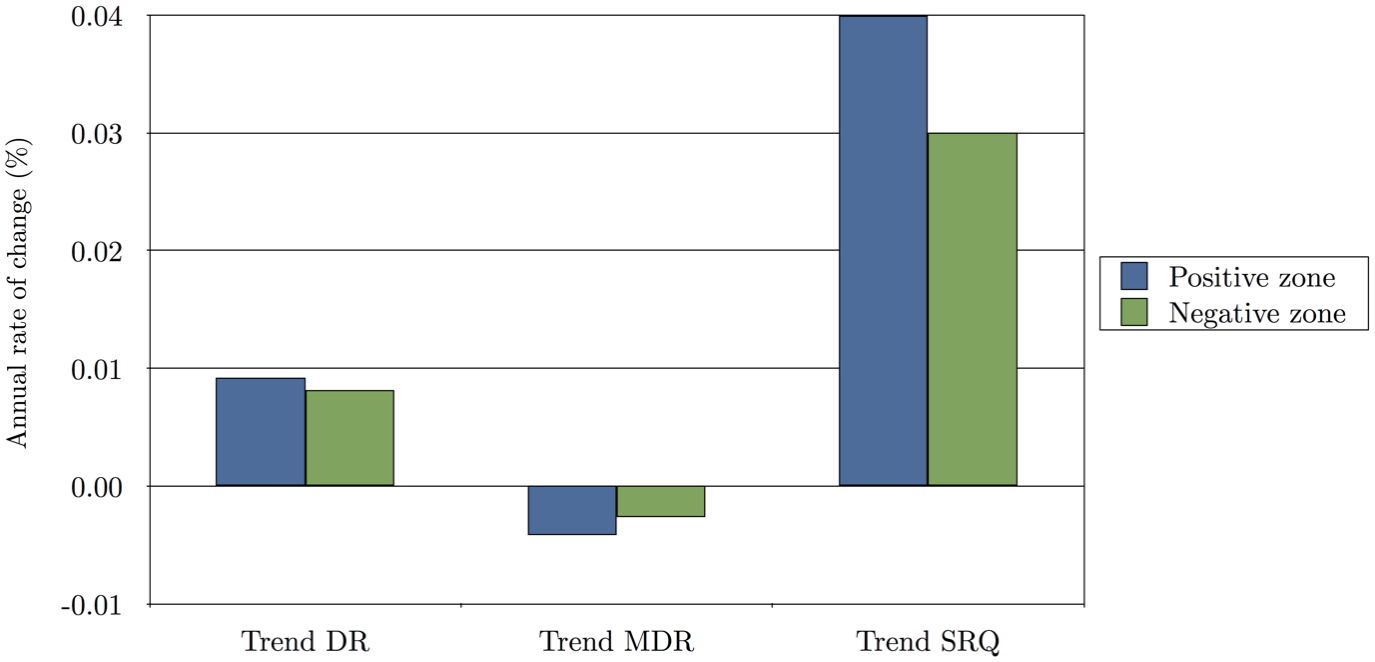
The annual change in developmental rates (DR), mortality rates of development (MRD), and survival rates of questing stages (SRQ) of H. marginatum in the period 1901–2009. Data were calculated and disaggregated for the areas where the tick is known to have permanent populations (positive zone: PZ) or where the tick has not yet been collected (negative zone: NZ).

The trends in DR, MRD and SRQ during the period 1901–2009 were disaggregated for the sites where the tick has been recorded as being present (PZ) or absent (NZ) (Fig 2). In the PZ, the percentage change in DR, MRD and SRQ was 0.092%, -0.042% and 0.04% per year, respectively. In the NZ, these values corresponded to 0.0082% (p = 0.005), -0.0026% (p = 0.005), and 0.03% (p<0.001) per year for the DR, MRD, and SRQ, respectively. Therefore, the rates of improvement in the critical physiological processes of the tick's life cycle were higher in the PZ than in the NZ.

**Fig 3:**
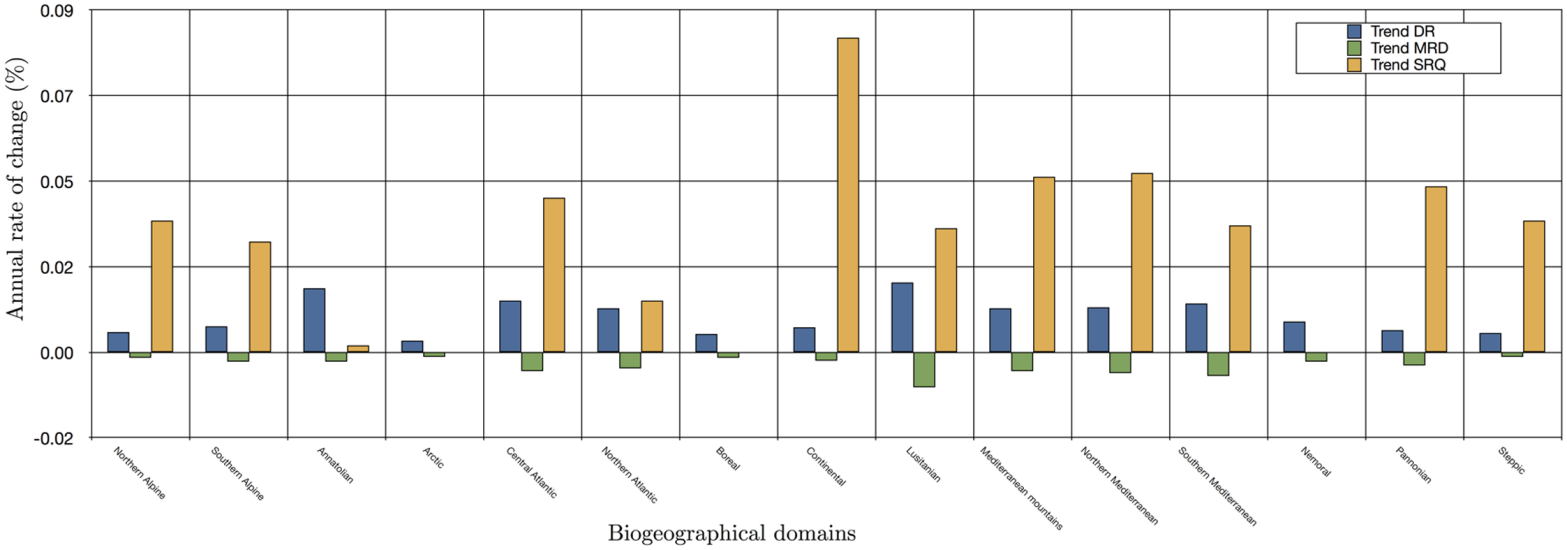
The annual change in developmental rates (DR), mortality rates of development (MRD), and survival rates of questing stages (SRQ) of *H*. *marginatum* in the period 1901–2009. Data were disaggregated for the climate domains outlined in Fig 1B.

The average DR, MRD and SRQ for *H*. *marginatum* calculated during 1901–2009 and disaggregated according to the biogeographic domains in the target territory are shown in Fig 3. The lowest values for DR and highest values for MRD were computed for areas with a cold climate (i.e., Alpine, Arctic, Boreal, Continental, Nemoral, and Pannonian). SRQ values were lowest for the Alpine, Arctic, Boreal, Continental, Nemoral, Pannonian, and Steppic areas. These results are consistent with the low temperature recorded in these domains, which promotes low development/questing rates and, therefore, high mortality rates. The trends of change were not the same for every domain. The calculated increase or decrease in the DR and MRD per year were larger in areas of the Anatolian (0.016% and 0.025%, respectively), Lusitanian (0.018% and 0.019%, respectively), and Mediterranean domains (0.012% and 0.006%, respectively). However, the SRQ increased more in the Continental and Atlantic domains, with growth of 0.08% per year and 0.045% per year, respectively.

**Fig 4:**
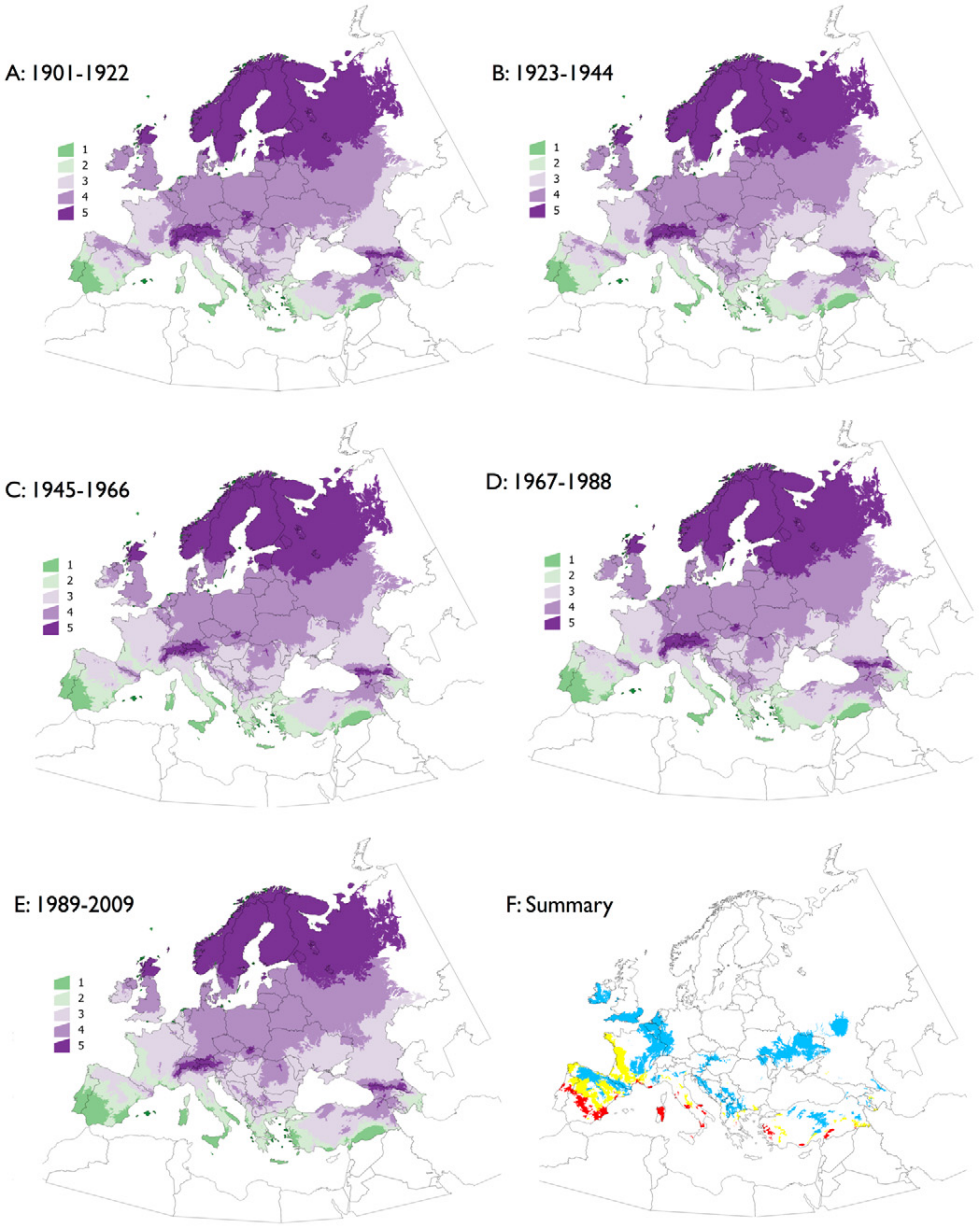
Classification of the target territory into categories of suitability for *H*. *marginatum*. The five time slices are shown in A-E classified according to the criteria in Table 1. A spatial summary of the rates of change for the physiological processes of *H*. *marginatum* during the complete period is included in F, in which blue indicates areas that switched from categories 4 and 5 to category 3; yellow indicates a switch from category 3 to 2; and red indicates a switch from category 2 to 1. The blue areas are territories where introduction of the tick without further development of permanent populations could be sporadically detected. The yellow areas are territories where conditions have changed enough to allow a positive turnover of the tick, if introduced. The red areas must be interpreted as sites where the tick is already established and conditions have improved, allowing the greatest population turnover.

Fig 4 spatially summarises all of the above results, producing a classification of the target territory into categories of suitability for *H*. *marginatum*, according to DR, MRD, and SRQ. In these maps, areas marked as 1 and 2 have optimal and suboptimal climate suitability, respectively, because they have the highest DR and SQR and lowest MRD. Categories 4 and 5 have the lowest DR and SRQ and highest MRD. The minimum conditions for tick survival are not supported in categories 4 and 5. Category 3 has intermediate characteristics, where ticks could occasionally survive or molt, and should be considered a border category. Major changes were observed in the western areas of the target territory, like the Iberian Peninsula and portions of France, and to a lesser extent in the western portions of central Europe. Almost all of Central Europe and Scandinavia remained unsuitable for the permanent survival of *H*. *marginatum* during the period 1901–2009, but zones of adequate suitability appeared in parts of the Atlantic coast of France. The largest improvement in climate suitability for the tick was observed during the periods 1967–1988 and 1989–2009.

**Fig 5:**
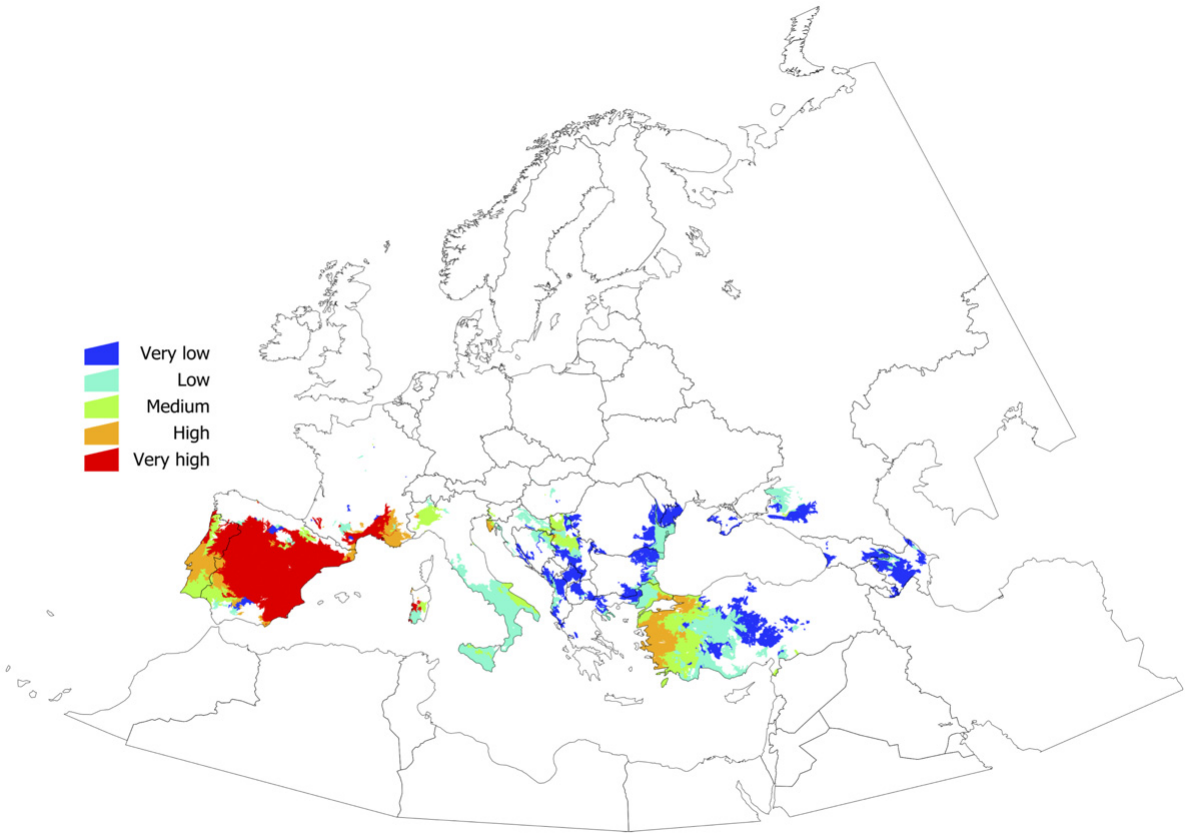
Evaluation of the target territory based on the trend of the change in the tick’s physiological processes over the period of study (exposure) and the density of domestic ruminants as hosts for adults (vulnerability). The map is intended to indicate the candidate areas where active surveys should be performed to check for changes in the tick’s population status.

Fig 5 shows a combined evaluation of exposure to the tick (because of its climate-driven fitness) and the vulnerability of the territory (because of the density of domestic ungulates). The figure shows, as five levels of risk, the areas of the target territory where changes in the population status of the tick should be expected. Such an evaluation should be interpreted with two different perspectives: if the tick already has permanent populations, these areas are suggestive of a high turnover of the tick populations; if these are territories where the tick has not yet been established, they should be interpreted as areas where, if introduced, tick populations could flourish because of adequate climate and host availability. In both cases, these territories should be considered candidate areas for active surveys of the tick to assess its presence or changes in density.

## Discussion

We assessed changes in the developmental and survival rates of *H*. *marginatum*, a tick important for public health, in a territory where its increased density or incidental introduction has been reported [37,40,41]. We further used these estimations to classify the target territory into categories, demonstrating that the climate improved the physiological processes of the tick along a latitudinal pattern during the period 1901–2009. The results presented in our assessment are objective and coherent because they were obtained using equations describing the life cycle processes. Therefore, the computed rates of change must be considered an objective evaluation of the impact of weather on the development and mortality of the tick, allowing a simple but realistic estimation of how climate trends impacted these processes over a period of 109 years. We assumed that the response of the tick to the climate was the same across its geographic range and that the tick tracks its climate niche, as has been tested for other animals [46,47], but this does not seem to be universal [48]. We also assumed that ticks do not habituate to new climate conditions, something that cannot be rejected by the empirical data available for the modeling framework. However, Pearson and Dawson [4] speculated that novel environments could expose genetic variation in fitness, on which the response to natural selection depends. Under these conditions of quick adaptation to newly appearing environments, the response may be less unpredictable. However, the phenotypic plasticity of ticks to the weather has not been explored.

The calculated rates of change in the life cycle processes of *H*. *marginatum* should not be directly translated into a higher abundance of ticks. Such rates of change may be greater in areas where the current climate suitability for the tick is very low, such as parts of Scandinavia, preventing its survival even at these unprecedentedly high rates of change. For a population persisting in a particular habitat, its mean absolute outcome must be adequate to replace itself over generations. Therefore, an adequate assessment must evaluate both the rates of change and the actual values for development and mortality to characterize the epidemiological status of an area and the expected turnover of the tick populations. Public health decision-making routinely requires the consideration of complex factors beyond the geographic distribution and determinants of disease risk [45]. Modeled results of the tick's physiological processes cannot be directly translated into maps of expected abundance. Thus, we assayed a classification of the target territory into simple categories. The strengths of this approach are not only its ability to help reconcile conflicting values and find a consensus decision among differing perspectives [49–52], but also its utility as a tool for understanding the issues at stake and inherent to the decision-making process itself [45].

Our results show a trend towards improved tick’s life cycle along a latitudinal gradient. However, the improvement is not linear in the time. The change was higher during the periods 1901–1922 and 1989–2009, and these findings are in line with other conclusions about the unprecedented rate of climate change in the last few decades [53–56]. The modeled impact of the climate on the developmental and survival rates of *H*. *marginatum* is higher in the area where the tick has been reported historically, probably because the trend towards higher temperature and lower relative humidity reported in the region [55,56]. However, results derived from the model predicted that large regions in the Atlantic domain might have experience increases in the development rate of up to 60%-80% and a more than 80% increase in the survival rates during developing and questing stages, which could be driven by a pronounced trend in temperature increase in the last 20–30 years [54–56]. These values are important because they refer to areas where the tick has been recorded sporadically during the past few years. This finding conciliates such records, which are probably accidental introductions, with claims of the effects of climate on the tick and contributes to supporting the affirmations that abiotic conditions are improving for *H*. *marginatum* in parts of Europe. Large areas in western and central Europe and the Balkans underwent significant improvements in the performance of *H*. *marginatum*, but at different rates. The DR and MDR are improving in Western Europe, whereas SQR is improving in areas of the Balkans, which is in line with previous reports [57]. Nevertheless, these improvements in the tick’s life cycle outcomes seem to be below the critical threshold necessary for the successful establishment of permanent tick populations given the criteria adhered to in this study. These trends seem to be operated by different drivers, i.e. while mean temperature is increasing in Western Europe, autumn and winters are consistently warmer in the last 15–25 years in the Balkans [57]. Our results have a point of uncertainty because the obvious simplifications assumed in the modeling approach. In example, no daylight-dependent diapause has been assumed (something that has not been addressed in the field) and we disregarded an estimation of abundance of the tick. The recorded increase of temperature in some regions could play a negative effect on the survival of questing stages. Interestingly, we only found partial spatial agreement between previous developments using correlative modeling [23,33,58] and the process-driven evaluations addressed in this paper, a point already addressed for other organisms [59].

The results suggest that the populations of *H*. *marginatum* may experience large changes in abundance at sites that have already been colonized, like most of the Iberian Peninsula, where a high abundance of hosts may produce a quick turnover of the population. The results also suggest that active surveys should be routinely carried out at specific points in Europe, where both weather conditions and host availability may provide an adequate background for the successful establishment of ticks in the case of introduction. However, we only included the density of domestic ungulates, which are common hosts for *H*. *marginatum* adults [41], as a driver of the estimation, but wild ungulates can feed large numbers of *Hyalomma* ticks [18]. A reliable map of the density of wild ungulates in a large territory like Europe is currently lacking. Because our estimation of areas to address with active surveys to estimate the turnover of *Hyalomma* ticks is driven by climate and domestic host availability, such an approximation could change after re-evaluation of the role, density, and movements of wild ungulates at such critical places in the target territory.

## Conclusions

This study demonstrated that the long-term climate trends have affected the rates of the life cycle processes of *H*. *marginatum*, and that the changes over the last few decades are unprecedentedly large. The risk of successfully establishing populations at northern latitudes seems to be low, even if the improvement in tick physiological processes has been exceptionally high at northern latitudes but still below a critical threshold. The present study resulted in a framework for assessing the spatial range that may be affected by the impact of climate on the life cycle rates of a health-threatening arthropod. The results produced a homogenizing framework that can incorporate data from a variety of sources and accommodate other vectorial systems; results are ascribed to recognized ecological regions over the target territory, facilitating description and categorization. There is an open field for investigating the effects of future climate scenarios on the physiological rates in the tick’s life cycle. Such active surveys and risk assessments are currently lacking for medically important ticks in the territory of reference.

## Supporting Information

S1 Text Equations used to calculate the development rates, mortality rates of development stages and survival rates of questing stages. (DOCX)

S2 Text R script with the equations to calculate the life cycle development, mortality and survival rates of *Hyalomma marginatum*. (DOCX)
