# Supplementary material for: The Impact of Climate Trends on a Tick Affecting Public Health: A Retrospective Modeling Approach for Hyalomma marginatum (Ixodidae)
Source: PLoS One. 2015 May 8;10(5):e0125760. doi: 10.1371/journal.pone.0125760 (PMC4425654; doi:10.1371/journal.pone.0125760)
Supplement: S1 Text — (DOCX) [file pone.0125760.s001.docx]

Text S1. **Equations used to calculate the development rates, mortality rates of development stages and survival rates of questing stages**. All the equations were fitted and validated by Estrada-Peña et al. (2011). For every equation, T is temperature in ºC and VD is average water deficit in mm.

1. Equations for development rates

Pre-oviposition: Tp = 50.775 - 1.0728T - 0.229VD (1)

Oviposition: To = 66.19 - 1.5638T - 0.179VD (2)

Incubation: Ti = 59.7 - 1.151T - 0.1014VD (3)

Molt of engorged nymphs to adults: Tm = 192.23 - 6.054T + 0.258VD (4)

where Tp, To, Ti and Tm are in days.

2. Equations for mortality rates of the developmental stages:

Females: Mf = 19.32 + 1.212T - 0.16VD (5)

Eggs: Me = 108.325 - 3.848T + 1.414VD (6)

Nymphs: Mn = 51.4786 + 1.525T - 0.22VD (7)

where Me and Mn are the mortality (in %) of either developing eggs or molting nymphs.

3. Equations for the survival of the questing stages:

Larvae: Ml = 16.1 + 0.814T - 0.21VD (8)

Adults: Ma = 14.3 + 0.792T - 0.14VD (9)

where Ml and Ma are the survival rates (in weeks) of the questing larvae or adults, respectively.
