# Supplementary material for: The Impact of Climate Trends on a Tick Affecting Public Health: A Retrospective Modeling Approach for Hyalomma marginatum (Ixodidae)
Source: PLoS One. 2015 May 8;10(5):e0125760. doi: 10.1371/journal.pone.0125760 (PMC4425654; doi:10.1371/journal.pone.0125760)
Supplement: S2 Text — (DOCX) [file pone.0125760.s002.docx]

**Text S2: R script with the equations to calculate the life cycle development, mortality and survival rates of *Hyalomma marginatum***.

#################################

# Life cycle for Hyalomma marginatum

# version running on decadal intervals of CRU temperature and water vapour values

##################################

library(popbio)

library(maptools)

library(sp)

library(plotrix)

avelarvae <- 50

aveadults <- 30

HD<-4

Actividad <- function (i) {

(0.203777*(HD^.114573))*((-.008*(i^2))+(0.336*(i))-2.128)

}

HembrasGordas <- function (i,j) {

30/((59.69858-(1.15089*(i))-(0.10136*(j)))+(50.77389-(1.07279*(i))-(0.22908*(j))))

}

HuevosGordos <- function(i,j){

30/(66.18899-(1.56382*(i))+(0.10136*(j)))

}

HembrasVivas <- function(i,j){

(108.3254-(3.718*(i))+(1.0139*(j)))/100

}

HuevosVivos <- function (i,j) {

(108.3254-(3.848*(i))+(1.4139*(j)))/100

}

NinfasGordas <- function (i,j) {

30/(192.2344-(6.054*(i))+(0.2577*(j)))

}

NinfasMuertas <- function (i,j) {

((51.4786+(1.002524*(i))-(0.222031*(j))))/100

}

puntacosTable <- read.csv("~/Desktop/ricinus_CRU/puntacosTable.csv")

resumen <- matrix(data=0, nc=7, nr=nrow(puntacosTable))

colnames(resumen)<-c("Sitio","Long", "Lat","Lambda Mean",

"Sum Dev","Sum Mort","Ratio Dev/Surv")

ClimateReal<-matrix(data=0, nc=27, nr=nrow(puntacosTable))

ClimateReal[,1] <- puntacosTable[,1]

ClimateReal[,2] <- puntacosTable[,2]

ClimateReal[,3] <- puntacosTable[,3]

pt <- readShapePoints("~/Desktop/ricinus_CRU/Puntacos.shp") ## The points for weather data

## Importing weather data

for (intervalo in seq(1901,2009,by=1))

{

print(intervalo)

x1 <- "~/Desktop/ricinus_CRU/temp/"

casilla <- 4

for (mes in 1:12)

{

x <- paste(x1,"cru_ts_3_10.1901.2009.tmp_",intervalo,"_",mes,".asc",sep="")

gr <- readAsciiGrid(x)

z <- overlay(gr, pt)

datos <- as.data.frame(z)

ClimateReal[,mes+3] <- datos[,1]/10

}

x1 <- "~/Desktop/ricinus_CRU/vap/"

for (mes in 1:12)

{

x <- paste(x1,"cru_ts_3_10.1901.2009.vap_",intervalo,"_",mes,".asc",sep="")

gr <- readAsciiGrid(x)

z <- overlay(gr, pt)

datos <- as.data.frame(z)

ClimateReal[,mes+15] <- datos[,1]/10

}

###### Main loop #######

for (bucle in 1:nrow(puntacosTable))

{

if (bucle%%100==0)

{

print(bucle*100/nrow(puntacosTable))

}

ClimateT <- matrix(data=0,nr=12,nc=1)

ClimateT[,1] <- ClimateReal[bucle,4:15]

LifeTable<-matrix(0, nc=16, nr=12)

colnames(LifeTable)<-c("DevEngFem","MortEngFem","DevEggs","MortEggs","GuessLarvae","% Larvae","Real Larvae","DevEngLarvae",

"MortEngLarvae","DevEngNymphs","MortEngNymphs","GuessAdults","% Adults","Real Adults","Lambda","Activity")

for (bucle2 in 1:12)

{

bucle3 <- bucle2+3

bucle4 <- bucle3+12

i <- ClimateReal[bucle,bucle3]

j <- ClimateReal[bucle,bucle4]

LifeTable[bucle2,1] <- HembrasGordas(i,j)

LifeTable[bucle2,2] <- HembrasVivas(i,j)

LifeTable[bucle2,3] <- HuevosGordos(i,j)

LifeTable[bucle2,4] <- HuevosVivos(i,j)

LifeTable[bucle2,8] <- NinfasGordas(i,j)

LifeTable[bucle2,9] <- NinfasMuertas(i,j)-(NinfasGordas(i,j)*0.85)

LifeTable[bucle2,10] <- NinfasGordas(i,j)

LifeTable[bucle2,11] <- NinfasMuertas(i,j)

LifeTable[bucle2,16] <- Actividad(i)

if (bucle2<6) LifeTable[bucle2,8]<-0 else LifeTable[bucle2,8]<-LifeTable[bucle2-2,10]

}

LifeTable[which(LifeTable[,1]>1),1] <- 1

LifeTable[which(LifeTable[,3]>1),3] <- 1

LifeTable[which(LifeTable[,2]<0),2] <- 0

LifeTable[which(LifeTable[,2]>1),2] <- 1

LifeTable[which(LifeTable[,16]<0),16] <- 0

LifeTable[which(LifeTable[,4]<0),4] <- 0

LifeTable[which(LifeTable[,4]>1),4] <- 1

LifeTable[which(LifeTable[,9]>1),9] <- 1

LifeTable[which(LifeTable[,11]>1),11] <- 1

LifeTable[which(ClimateT[,1]<6),1] <- 0

LifeTable[which(ClimateT[,1]<6),3] <- 0

LifeTable[which(ClimateT[,1]<6),8] <- 0

LifeTable[which(ClimateT[,1]<6),10] <- 0

# # Recruitment by development

acumula<-0; puntero<-1; volvedor<-1

repeat

{

while (acumula<1)

if (puntero>nrow(ClimateT)) break

else {acumula<-acumula+LifeTable[puntero,1]; puntero<-puntero+1}

if (puntero>nrow(ClimateT)) break

else LifeTable[volvedor,5]<-puntero; acumula<-0; puntero<-volvedor+1; volvedor<-volvedor+1

}

acumula<-0; puntero<-1; volvedor<-1

repeat

{

while (acumula<1)

if (puntero>nrow(ClimateT)) break

else {acumula<-acumula+LifeTable[puntero,8]; puntero<-puntero+1}

if (puntero>nrow(ClimateT)) break

else LifeTable[volvedor,12]<-puntero; acumula<-0; puntero<-volvedor+1; volvedor<-volvedor+1

}

# # Self-protection against overfitting

acumula<-0

for (linea in 1:nrow(LifeTable))

{

acumula<-acumula+LifeTable[linea,3]

if (LifeTable[linea,10]<0) LifeTable[linea,10]<-0

}

minimLarvae <- min(LifeTable[which(LifeTable[,5]>0),5])

for (linea in 1:nrow(LifeTable))

{

if (LifeTable[linea,5]==0) LifeTable[linea,5] <- minimLarvae

}

minimAdults <- min(LifeTable[which(LifeTable[,12]>0),12])

for (linea in 1:nrow(LifeTable))

{

if (LifeTable[linea,12]==0) LifeTable[linea,12] <- minimAdults

}

for (linea in 1:nrow(LifeTable))

{

LifeTable[LifeTable[linea,5],6] <- LifeTable[LifeTable[linea,5],6]+1

LifeTable[LifeTable[linea,12],13] <- LifeTable[LifeTable[linea,12],13]+1

}

LifeTable[,7] <- c(LifeTable[,6]*LifeTable[,2]*LifeTable[,4]*LifeTable[,16])

LifeTable[,6] <- c((rescale(LifeTable[,7],0:1))*avelarvae)

LifeTable[,14] <- c(LifeTable[,13]*LifeTable[,8]*LifeTable[,10]*LifeTable[,16])

LifeTable[,13] <- c((rescale(LifeTable[,14],0:1))*aveadults)

Building matrixes for eigen analyis and lambda (5 x 5).

for (linea in 1:nrow(LifeTable))

{

container<-matrix(0, nr=5, nc=5)

container[1,5]<-2000

container[2,1]<-LifeTable[linea,1];container[2,2]<-(1-LifeTable[linea,2])

container[3,2]<-LifeTable[linea,3];container[3,3]<-(1-LifeTable[linea,4])

container[4,3]<-LifeTable[linea,8];container[4,4]<-(1-LifeTable[linea,9])

container[5,4]<-LifeTable[linea,10];container[5,5]<-(1-LifeTable[linea,11])

LifeTable[linea,15]<-lambda(container)-1

LifeTable[which(LifeTable[,15]<0),15] <- 0

}

resumen[bucle,1] <- puntacosTable[bucle,1]

resumen[bucle,2] <- puntacosTable[bucle,2]

resumen[bucle,3] <- puntacosTable[bucle,3]

resumen[bucle,4] <- mean(LifeTable[,15])

resumen[bucle,5] <- sum(LifeTable[,1]+LifeTable[,3]+LifeTable[,10])

resumen[bucle,6] <- sum(LifeTable[,2]+LifeTable[,4]+LifeTable[,11])

resumen[bucle,7] <- resumen[bucle,5]/resumen[bucle,6]

#### End of main loop ####

}

resultados <- c(paste("resultados_",intervalo,".csv",sep=""))

setwd("~/Desktop/assessment_Hyalomma/")

write.csv(resumen, file = resultados, quote = TRUE,eol = "\n")

}
